# α-Synuclein in blood exosomes immunoprecipitated using neuronal and oligodendroglial markers distinguishes Parkinson’s disease from multiple system atrophy

**DOI:** 10.1007/s00401-021-02324-0

**Published:** 2021-05-15

**Authors:** Suman Dutta, Simon Hornung, Adira Kruayatidee, Katherine N. Maina, Irish del Rosario, Kimberly C. Paul, Darice Y. Wong, Aline Duarte Folle, Daniela Markovic, Jose-Alberto Palma, Geidy E. Serrano, Charles H. Adler, Susan L. Perlman, Wayne W. Poon, Un Jung Kang, Roy N. Alcalay, Miriam Sklerov, Karen H. Gylys, Horacio Kaufmann, Brent L. Fogel, Jeff M. Bronstein, Beate Ritz, Gal Bitan

**Affiliations:** 1 Department of Neurology, David Geffen School of Medicine, University of California, Los Angeles, CA 90095 USA; 2 Department of Epidemiology, Fielding School of Public Health, University of California, Los Angeles, CA 90095 USA; 3 Department of Medicine, Division of General Internal Medicine and Health Services Research, David Geffen School of Medicine, University of California, Los Angeles, CA 90095 USA; 4 Department of Neurology, Dysautonomia Center, The Marlene and Paolo Fresco Institute for Parkinson’s and Movement Disorders, New York University School of Medicine, New York, NY 10016 USA; 5 Banner Sun Health Research Institute, Sun City, AZ 85351 USA; 6 Mayo Clinic College of Medicine, Mayo Clinic Arizona, Scottsdale, AZ 85259 USA; 7 Institute for Memory Impairments and Neurological Disorders, University of California, Irvine, CA 92697 USA; 8 Department of Neurology, Taub Institute for Research on Alzheimer’s Disease and the Aging Brain, Columbia University, New York, NY 10032 USA; 9 Department of Neurology, University of North Carolina School of Medicine, Chapel Hill, NC 27599 USA; 10 School of Nursing, University of California, Los Angeles, CA 90095 USA; 11 Clinical Neurogenomics Research Center, David Geffen School of Medicine, University of California, Los Angeles, CA 90095 USA; 12 Brain Research Institute, University of California, Los Angeles, CA 90095 USA; 13 Molecular Biology Institute, University of California, Los Angeles, CA 90095 USA; 14 Present Address: Division of Peptide Biochemistry, Technical University of Munich, 85354 Freising, Germany

**Keywords:** Biomarker, Extracellular vesicles, Synucleinopathy, Biofluid

## Abstract

The diagnosis of Parkinson’s disease (PD) and atypical parkinsonian syndromes is difficult due to the lack of reliable, easily accessible biomarkers. Multiple system atrophy (MSA) is a synucleinopathy whose symptoms often overlap with PD. Exosomes isolated from blood by immunoprecipitation using CNS markers provide a window into the brain’s biochemistry and may assist in distinguishing between PD and MSA. Thus, we asked whether α-synuclein (α-syn) in such exosomes could distinguish among healthy individuals, patients with PD, and patients with MSA. We isolated exosomes from the serum or plasma of these three groups by immunoprecipitation using neuronal and oligodendroglial markers in two independent cohorts and measured α-syn in these exosomes using an electrochemiluminescence ELISA. In both cohorts, α-syn concentrations were significantly lower in the control group and significantly higher in the MSA group compared to the PD group. The ratio between α-syn concentrations in putative oligodendroglial exosomes compared to putative neuronal exosomes was a particularly sensitive biomarker for distinguishing between PD and MSA. Combining this ratio with the α-syn concentration itself and the total exosome concentration, a multinomial logistic model trained on the discovery cohort separated PD from MSA with an AUC = 0.902, corresponding to 89.8% sensitivity and 86.0% specificity when applied to the independent validation cohort. The data demonstrate that a minimally invasive blood test measuring α-syn in blood exosomes immunoprecipitated using CNS markers can distinguish between patients with PD and patients with MSA with high sensitivity and specificity. Future optimization and validation of the data by other groups would allow this strategy to become a viable diagnostic test for synucleinopathies.

## Introduction

Synucleinopathies are neurodegenerative diseases characterized by abnormal accumulation of intracellular α-syn aggregates. In Parkinson’s disease (PD) and dementia with Lewy bodies (DLB), α-syn accumulates in intraneuronal Lewy bodies and Lewy neurites, whereas in multiple system atrophy (MSA), α-syn deposits primarily as glial cytoplasmic inclusions (GCIs) in oligodendrocytes [8, 45, 67]. Accumulation and deposition of α-syn also occur in other neurodegenerative diseases and conditions, such as Alzheimer’s disease (AD), pure autonomic failure, rapid eye movement sleep behavioral disorders, traumatic brain injury, and neuroaxonal dystrophies [29, 41, 44]. Though synucleinopathies have distinct symptoms and underlying pathophysiology, during early stages, they often are misdiagnosed due to overlapping symptoms with other synucleinopathies, atypical parkinsonian tauopathies, spinocerebellar ataxias, and/or dementias [18, 46, 48].

The diagnosis accuracy of PD is approximately 80% and is lower in new cases [1, 4]. The rates of misdiagnosis are higher for the rarer synucleinopathies [57, 62]. A retrospective post-mortem study found that about one in four patients who received the clinical diagnosis of PD by a general neurologist actually was a misdiagnosis of MSA or progressive supranuclear palsy (PSP) [33]. A separate autopsy study of 134 patients diagnosed with MSA found 71% accuracy in patients diagnosed with probable MSA and 60% in those diagnosed with possible MSA [34].

The clinical diagnosis of MSA currently is based on sets of autonomic and motor symptoms that provide variable degrees of diagnostic certainty [23, 68]. Compared to PD, patients with MSA have more severe, generalized, and rapidly progressive autonomic failure, particularly in the early stages of disease. Thus, autonomic testing can be helpful to assist in the diagnosis of MSA [50, 51]. For example, abnormal thermoregulatory sweat testing has a high specificity for distinguishing MSA from PD or DLB. Preserved myocardial ^123^I- Iobenguane imaging is a useful supporting feature of MSA, as it is typically impaired in PD and DLB, and an elevated post-void bladder residual volume, particularly in early stages, is highly indicative of MSA [51]. However, autonomic dysfunction also can be a confounder [49]. For example, the presence of neurogenic orthostatic hypotension is not useful for discriminating MSA from PD as it can be present in all synucleinopathies [38, 50].

Multiple attempts to develop imaging and cerebrospinal fluid (CSF)-based biomarkers for synucleinopathies [10, 56, 59] have been made to date, but due to their lack of specificity and/or invasive nature, none has translated yet into regular clinical practice. Currently, a definite diagnosis can only be made at autopsy [23, 55]. Development of sensitive and specific biomarkers for synucleinopathies therefore is an urgent public health need for proper disease diagnosis, establishment of appropriate inclusion and exclusion criteria for clinical trials, and use of pharmacodynamic measures of treatment effects.

Extracellular vesicles (EVs), including exosomes and ectosomes, are heterogeneous vesicles released by virtually all cell types. They contain parent-cell-specific cargoes of proteins, lipids, and nucleic acids [63]. Exosomes are the smallest and most abundant EVs. They provide an important mode of intercellular communication and a rich source of biomarkers [31, 39, 53]. Recent studies have shown that exosomes were involved in interneuronal and neuron–glia communication [6, 20, 21]. α-Syn has been shown to transfer via exosomes among different brain cells [3, 12], seed aggregation [27], and induce apoptosis in recipient cells [13, 15].

Recently, several studies have demonstrated that exosomes isolated from serum or plasma by immunoprecipitation using neuronal markers carried cargo from their putative cells of origin through the blood–brain barrier and thus could provide a “window” into pathologic processes in the brain, offering a major advancement in analyzing brain biomarkers non-invasively [17, 24, 25, 60, 61]. Although over 50 papers using this strategy have been published since 2014, to date, no study has demonstrated unequivocally that the origin of the exosomes indeed was CNS neurons. Therefore, we refer to these exosomes as putatively originating in the CNS.

Shi et al. [61] have reported a significantly higher concentration (~ twofold) of α-syn in putative neuronal exosomes isolated from the plasma of patients with PD compared to healthy individuals, yet a substantial overlap was found between the groups. More recently, Jiang et al. [32] used a combination of α-syn and clusterin concentrations measured in putative neuronal exosomes and found that they separated efficiently patients with PD from those with atypical parkinsonian syndromes. The study included 14 patients with MSA in which the putative neuronal exosomal α-syn concentrations were significantly lower than in patients with PD. Yu et al. measured α-syn concentrations in putative neuronal and oligodendroglial exosomes, the latter isolated by immunoprecipitation from patient plasma using the oligodendrocyte marker 2,3-cyclic nucleotide-3-phosphodiesterase (CNPase), as a potential diagnostic biomarker for separating patients diagnosed with PD (*N* = 34) from those diagnosed with MSA (*N* = 32) [70]. α-Syn concentrations again were found to be lower in the exosomes from patients with MSA than in those with PD, yet the differences were small and the degree of overlap between the groups was high.

Because α-syn accumulates primarily in neurons in PD and oligodendrocytes in MSA, we hypothesized that comparing its levels in putative neuronal and oligodendroglial exosomes might allow differentiating MSA from PD. We used a different marker from the one chosen by Yu et al., myelin oligodendrocyte glycoprotein (MOG), for isolation of oligodendroglial exosomes. Using this marker, we found in both diseases a significant elevation of α-syn concentration in putative oligodendroglial exosomes compared to healthy controls. α-Syn concentrations were particularly high in the MSA group compared to the control and PD groups. The ratio between α-syn concentrations in putative oligodendroglial versus neuronal exosomes was found to be a sensitive biomarker for distinguishing between PD and MSA.

## Materials and methods

### Study populations

#### Discovery cohort

Serum samples were collected initially for a discovery cohort from 51 healthy controls, 50 patients with PD, and 30 patients with MSA. The sample sources for this cohort included: (1) the Parkinson’s Environment and Genes (PEG) study, UCLA; (2) the Institute for Memory Impairments and Neurological Disorders, UC Irvine; (3) the Clinical Neurogenomics Research Center, UCLA; and (4) the Dysautonomia Center, New York University School of Medicine. Except for one sample from NYU, the control samples were collected as part of the PEG study at the same time of collecting patient samples and were primarily from patient spouses or other relatives. After discovering that samples stored for > 5 years had reduced signal (Supplementary Information), all of these samples were replaced with new samples stored < 5 years. The final composition of the discovery cohort is summarized in Table 1. Blood-collection procedures were approved by the respective Human Subjects Committees at each institution and informed consent was obtained from all participants.

**Table 1:** Demographic and clinical data for final discovery cohort

	All samples	Control	PD	MSA (C:P)
Total samples	131	50	51	30 (26:4)
Age^a^ (range)	66.3 ± 11.1 (35–90)	63.2 ± 12.2 (35–86)	71.5 ± 9.5 (37–90)	62.7 ± 8.3 (46–79)
Sex (male:female)	67:64	22:28	32:19	13:17
Disease duration in years^a^ (range)	7.0 ± 4.7 (0–19)		8.4 ± 5.0 (0–19)	4.4 ± 2.6 (0–10)
Race/ethnicity^b^	As^c^ – 8	As – 1	As – 0	As – 7
	B – 2	B – 0	B – 1	B – 1
	H – 6	H – 4	H – 1	H – 1
	HN – 14	HN – 6	HN – 8	HN – 0
	NA – 3	NA – 2	NA – 1	NA – 0
	ND – 2	ND –1	ND –0	ND –1
	W – 96	W – 36	W – 40	W – 20
UPDRS^d^ motor (range)			25.4 ± 15.3 (0–59)	
H&Y^d^ (range)	2.7 ± 1.1 (1–5)		2.5 ± 1.0 (1–5)	3.8 ± 1.0 (2–5)
MMSE^d^ (range)			26.5 ± 6.2 (0–30)	26.5 ± 9.3 (10–30)^e^

^a^Mean ± SD. ^b^As—Asian; B—Black; H—Hispanic; HN—Hispanic, non-White; NA—Native American, ND—non-disclosed; W—White. ^c^Korea—2, Philippines—1, Taiwan—1, Vietnam—1, Undefined Asian—3. ^d^UPDRS—Unified Parkinson’s disease rating scale, H&Y—Höhn and Yahr rating scale, MMSE—Mini-Mental State Examination. ^e^Converted from Montreal Cognitive Assessment (MoCA) according to Lawton et al. [36]

#### Postmortem cohort

Serum samples collected post-mortem with post-mortem interval (PMI) 1.5–6 h from 49 patients with PD and 12 patients with MSA were obtained from Banner Sun Health Research Institute. The diagnosis in this cohort was validated pathologically in each case as described previously [5].

#### Validation cohort

Serum or plasma samples were collected from 50 healthy controls, 50 patients with PD, and 50 patients with MSA. Each sample was from a unique donor. The samples sources for this cohort were similar to the discovery cohort, with an addition of two additional sources: The Department of Neurology, Columbia University and the Easton Center biobank at UCLA. About half of the control samples were again from the PEG study, whereas the other half were from Columbia and were collected as part of a different project from the one supplying the MSA samples. After discovering that samples stored for > 5 years had reduced signal (Supplementary Information), all of these samples were replaced with new samples stored < 5 years. The final composition of the validation cohort is summarized in Table 2.

**Table 2:** Demographic and clinical data for final validation cohort

	All Samples	Control	PD	MSA (C: P:mixed)
Total samples	154	51	53	50 (33:13:4)
Age^a^ (range)	67.3 ± 9.8 (40–88)	66.6 ± 8.9 (44–88)	72.0 ± 10.2 (40–88)	62.9 ± 7.9 (47–79)
Sex (male:female)	82:72	23:28	33:20	26:24
Disease duration in years^a^ (range)	6.9 ± 4.1 (1–26)		8.1 ± 3.8 (2–20)	5.6 ± 4.0 (1–26)
Race/ethnicity^b^	As^c^ – 8	As – 1	As – 1	As – 6
	B – 4	B – 0	B – 0	B – 4
	H – 22	H – 10	H – 11	H – 1
	HN – 0	HN – 0	HN – 0	HN – 0
	NA – 5	NA – 1	NA – 4	NA – 0
	ND – 6	ND –4	ND –1	ND –1
	W – 109	W – 35	W – 36	W – 38
UPDRS^d^ motor (range)			26.0 ± 13.3 (0–49)	
H&Y^d^ (range)	2.3 ± 0.9 (0–5)		2.3 ± 0.8 (0–4)	3.0 ± 2.8 (1–5)
MMSE^d^ (range)			27.6 ± 2.3 (18–30)	28.0 ± 1.7 (24–30)^e^

^a^Mean ± SD. ^b^As—Asian; B—Black; H—Hispanic; HN—Hispanic, non-White; NA—Native American, ND—non-disclosed; W—White. ^c^Undefined Asian—8. ^d^UPDRS—Unified Parkinson's disease rating scale, H&Y—Höhn and Yahr rating scale, MMSE—Mini-Mental State Examination. ^e^Converted from Montreal Cognitive Assessment (MoCA) according to Lawton et al. [36]

#### PD diagnosis

Board-certified neurologists diagnosed PD according to the Movement Disorders Society clinical diagnostic criteria for PD [52]. At baseline and each follow-up, UCLA movement disorder specialists (providing 93% of the PD samples used in this study) confirmed a diagnosis of idiopathic PD and evaluated motor features using the Unified Parkinson’s Disease Rating Scale (UPDRS parts I, III, and IV) and Höhn and Yahr (H&Y) staging. At each time point, > 80% of the participants were evaluated in an ‘off’ (≥ 12 h) medication state. For those ‘on’, a correction factor was added to their UPDRS-III total score, equal to the mean difference of ‘off’ and ‘on’ scores in all patients. The average of the whole sample also was used to impute missing items (mainly due to disability impeding evaluation of specific items such as ‘arise from chair’). The MDS version of the UPDRS-III was adopted in 2016, and thus, scores derived from this scale were corrected by subtracting seven points according to the method of Hentz et al. [30]. Samples provided by NYU were all from patients off medication and the patients were evaluated using the MDS version of the UPDRS-III. Cognitive function was assessed using the Mini-Mental State Examination (MMSE).

#### MSA diagnosis

Patients diagnosed with MSA fulfilled current consensus criteria for possible or probable MSA [23]. Detailed information was collected on demographics, risk factors, chronic diseases, and medication use. Patients were assessed using the Unified Multiple System Atrophy Rating Scale (UMSARS) or Scale for Assessment and Rating of Ataxia (SARA). Whenever possible, patients were examined off antiparkinsonian medications. Cognitive function was assessed using the MMSE or Montreal Cognitive Assessment (MoCA).

#### Serum collection

Peripheral blood from living persons was drawn by venipuncture using a BD Vacutainer push-button blood-collection kit and left to coagulate in silicone-coated serum-collection tubes for 15–20 min. After centrifugation at 1500*g* for 15 min at 4 °C, the serum was collected and either processed immediately or aliquoted and stored at − 80 °C.

Postmortem serum was obtained as described previously [5]. Briefly, blood was drawn from the left ventricle by a transthoracic puncture using 30-mL, disposable, polyethylene syringes fitted with 8-cm long, 18-gauge needles. Serum was separated from the blood using standard serum separator vacuum tubes (7 mL) prior to 10 min centrifugation, aliquoted into 0.5-mL polyethylene microcentrifuge tubes, and stored at − 80 °C.

#### Plasma collection

Plasma samples were obtained as described previously [2]. Briefly, peripheral blood was drawn by venipuncture and collected into EDTA tubes. Within 30 min of collection, the plasma tubes underwent centrifugation at 4 ºC for 15 min at 1500*g*. The plasma then was aliquoted into 0.5 mL aliquots (control samples) or 1.0 mL aliquots (MSA samples) and stored at − 80 ºC.

#### Measurement of serum hemoglobin

Hemoglobin concentration was measured using a hemoglobin assay kit (Sigma-Aldrich) according to the manufacturer’s instructions. Fifty microliters of undiluted serum/plasma samples were used, and hemoglobin concentration was quantified with reference to a standard curve generated using freshly prepared stock hemoglobin.

#### EV isolation

Frozen serum or plasma samples were thawed on ice. Protease and phosphatase inhibitor cocktails (PPi, Sigma-Aldrich) were added immediately, and the samples were centrifuged at 2000*g* for 10 min at 4 °C to precipitate any cells or cell-debris remnants. Clear supernates (250 μL) then were mixed gently with 63 μL of an ExoQuick Exosome Precipitation Solution (System Biosciences) and incubated on ice for 1 h, followed by centrifugation at 1500*g* for 30 min at 4 °C. The resulting pellets were suspended in pre-chilled PBS containing 1% (*w*/*v*) bovine serum albumin (BSA) and PPi for subsequent enrichment steps.

#### Exosome concentration measurement

Exosome concentration was measured indirectly using the ExoELISA Ultra CD81 assay (System Biosciences). After isolation, exosomes were resuspended in PBS supplemented with PPi. Five hundred μg of total protein of each sample and CD81 standards were loaded onto a 96-well plate and incubated for 1 h at 37 °C. The wells then were washed thrice for 5 min and incubated with an anti-CD81 primary antibody in blocking buffer with gentle agitation at room temperature (RT). Wells then again were washed thrice for 5 min and incubated with a horseradish peroxidase (HRP)-conjugated secondary antibody in blocking buffer with gentle agitation at RT for 1 h. Then, 50 μL of tetramethylbenzidine (TMB) substrate were added to each well and incubated for 10 min with shaking at RT. Stop buffer was added and absorbance was measured at 450 nm using a Synergy HTX plate reader (BioTek, USA).

#### Immunoprecipitation (IP) of exosomes using neuronal and oligodendroglial markers

Two μg each of anti-L1 cell-adhesion molecule (L1CAM, clone 5G3, Santa Cruz Biotechnology) for enrichment of neuronal exosomes, anti-myelin oligodendrocyte glycoprotein (MOG, clone D-2, Santa Cruz Biotechnology) for enrichment of oligodendroglial exosomes, or normal mouse IgG (Life Technologies) as a negative control were used to coat 1 mg of M-270 epoxy Dynabeads using a Dynabeads Antibody Coupling Kit (Life Technologies) overnight at 37 °C with gentle rotation following the manufacturer’s instructions. Antibody-coated beads then were mixed gently with the isolated serum/plasma EVs in chilled phosphate-buffered saline (PBS), pH 7.4, containing 1% (*w*/*v*) BSA and PPi and incubated overnight at 4 °C with gentle rotation. The bead-attached exosomes then were washed with 1 mL of 0.1% (*w*/*v*) BSA in PBS, pH 7.4, and transferred into new tubes in which the exosomes were lysed by incubating in 25 μL of radioimmunoprecipitation assay (RIPA) buffer (Thermo-Fisher Scientific) containing PPi for 10 min at room temperature and stored at − 80 °C.

#### On-bead flow-cytometry analysis of CD9+ exosome complexes

EVs precipitated from pooled human serum by the ExoQuick kit were resuspended in 1% (*w*/*v*) BSA in PBS and incubated with magnetic beads conjugated to anti-L1CAM or anti-MOG antibodies, as described above. As a negative control, the same beads were incubated with 1% (*w*/*v*) BSA in PBS in the absence of exosomes. After the incubation, the beads were washed twice in cold PBS and resuspended in 200 μL of exosome-stain buffer (system Biosciences). Five microliters of FITC-conjugated anti-human CD9 antibody (clone SN4 C3 3A2, eBioscience, USA) were added and incubated for 2 h at 4 °C with gentle rotation. Following washing, the beads were resuspended in 500 μL of wash buffer (System Biosciences) and 5000 events were counted using a BD LSR II flow-cytometry instrument (BD Biosciences, USA). The data were analyzed using FlowJo software.

#### Microfluidic resistive pulse sensing

MRPS measurements were performed using an nCS1 instrument (Spectradyne, USA) equipped with disposable TS-300 polydimethylsiloxane cartridges. To generate an appropriate ionic electrical current in the analyte, 1% (*w*/*v*) BSA in PBS, pH 7.4, was used as a running buffer. Three µL of sample were used for measurement and ≥ 1000 particles were counted per analysis. Calibration was performed using calibration beads and data were analyzed using nCS1 Data Analyzer (Spectradyne). Filters were applied for data analysis to exclude false-positive signals. The filters excluded detected particle events characterized by user-defined signal-to-noise ratio, transit time, particle diameter or peak symmetry.

#### TEM analysis of immunoprecipitated exosomes

Exosomes were eluted from anti-L1CAM- or anti-MOG-coated beads using 50 µL of exosome-elution buffer (System Biosciences), mixed with 50 μL 2% (*v*/*v*) paraformaldehyde (PFA) in PBS, and incubated for 20 min. Formvar carbon-coated grids (FCF400-CU, Electron Microscopy Sciences) were glow-discharged on a Pelco easiGlow instrument (Ted Pella, Inc.) for 2 min. Twenty μL of the fixed-exosome solution were placed on the grid and incubated for 20 min at RT. The grids then were washed thrice by floating them upside down on a 100-µL drop of filtered, deionized water (Milli-Q, Millipore). Exosomes were further fixed on the grids in 20 μL of 1% (*v*/*v*) glutaraldehyde for 5 min and stained with 2% (*w*/*v*) uranyl acetate for 10 min. The grids were washed twice in deionized water and imaged using a JEOL JEM-1200 EX transmission electron microscope operated at an acceleration voltage of 80 kV at a magnification of 80,000 × .

#### ECLIA measurement of α-syn

α-Syn concentration was measured using a U-PLEX Human α-Synuclein Kit (Meso Scale Discovery) according to the manufacturer’s instructions. The measurements were done by users blinded to diagnosis, demographic data, or any other identifying information. Briefly, a biotinylated anti-human α-syn capture antibody was added to small-spot streptavidin-coated wells and incubated at room temperature with shaking at 800 rpm for 1 h. After washing the wells thrice with PBS containing 0.5% (*v*/*v*) Tween-20, samples and calibration standards were added along with a Sulfo-TAG-conjugated anti-human α-syn detection antibody and incubated at room temperature with shaking at 800 rpm for 2 h. After washing the wells thrice, read buffer was added and the plates were read using MSD Sector Imager (Model-1250) or QuickPlex SQ 120 instruments. The data were analyzed using Discovery Workbench 4.0 software and quantified with reference to a freshly prepared α-syn standard curve.

#### Analysis of L1CAM in fractionated serum/plasma

Size-exclusion columns (35 nm, qEVoriginal, Izon sciences) were used to fractionate pooled serum or plasma samples (Innovative Research, Novi, MI). According to the manufacturer’s instructions, exosomes elute in fractions 6–10, whereas fractions 11–17 contain free proteins and other smaller molecules in the serum/plasma. The eluted fractions were analyzed using an L1CAM ELISA kit (Millipore-Sigma, St. Louis, MO) according to manufacturer’s instructions. Briefly, the samples and human L1CAM protein standards were added to the capture-antibody-coated wells and incubated for 2.5 h at room temperature. After washing the wells four times, biotinylated anti-L1CAM detection antibody was added to the wells and incubated for 1 h. Wells then were washed four times and incubated with HRP-conjugated streptavidin for 45 min at room temperature with gentle agitation. After washing the wells, TMB substrate was added and further incubated for 30 min in the dark with gentle agitation. Finally, a stop solution was added and the wells were read at 450 nm immediately.

#### Statistical analysis

For all descriptive analyses, categorical variables were expressed as frequency (percentage), whereas continuous variables were expressed as median (interquartile range). Baseline variables were compared between the training and the validation cohorts separately for each group using the Chi-square or Fisher’s tests for comparisons involving categorical variables and the Wilcoxon rank sum test for comparisons involving continuous variables. Each of the individual biomarkers were compared across groups using the Kruskal–Wallis test. Correlations across individual biomarkers were evaluated using the Spearman method. Multivariable models for predicting diagnosis status based on multiple biomarkers combined were developed in the discovery cohort dataset using the multinomial logistic model with LASSO variable selection. Prediction accuracy for each pairwise combination of groups was performed using receiver-operating characteristic (ROC) analysis based on the above logistic model in the discovery cohort and in the validation cohort. Reported are the area under the curve (AUC), the sensitivity, and the specificity, which were evaluated at the best threshold, defined as the value of the linear predictor in the logistic model which maximized the unweighted sum of the sensitivity and the specificity. Multivariable analyses were performed using four additional classifiers including the linear discriminant analysis [11], quadratic discriminant analysis [11], classification tree for binary recursive partitioning [9], and K-nearest neighbor [42]. The prediction accuracies of each model were evaluated using the method by Hand and Till [26]. For the PD and MSA groups, probable vs possible diagnosis, and for MSA-C vs MSA-P, biomarkers were compared using the Wilcoxon rank sum test. The correlation between individual biomarkers and clinical measures of progression were evaluated using the Spearman method. The associations between selected measures of progression and multiple biomarkers combined were evaluated using linear regression models as sample size permitted. Analyses were performed using Prism 8.4 (GraphPad, San Diego, CA) or R version 4.0.2 (Copyright © 2020 The R Foundation for Statistical Computing).

## Results

### Inclusion strategy

Unlike biomarker studies that emphasize strict control of sample collection protocols and matching among groups and cohorts, we used more lenient inclusion criteria in an attempt to obtain a better representation of the high variability in the patient population and in various clinical settings. Both the discovery and validation cohorts included samples collected either in the field in a population-based study or in university-hospital-based clinics. Patients with PD were characterized by the diagnosing specialists as definite, probable, or possible PD and patients with MSA included both probable and possible diagnosis. In each MSA category, both the cerebellar type (MSA-C) and parkinsonian type (MSA-P) were included. In the discovery cohort, all the samples were serum, whereas in the validation cohort, ~ 40% of the samples were plasma.

A detailed description of the exosome isolation, enrichment, origin validation, assay reproducibility, and limitation of sample storage period is provided as Supplementary Results and Supplementary figs. 1–5, online resources.

### Discovery cohort

Before immunoprecipitation of exosomes using CNS biomarkers, we asked whether the total number of exosomes or the total concentration of α-syn in the serum samples differed among the groups. The number of exosomes can be estimated conveniently using a commercial CD81 ELISA kit in which the CD81 signal is converted to exosome concentration. To improve normalization of the data for statistical analysis, here and in all subsequent analyses, the values were log-transformed and are presented as log values in the figures. However, the untransformed values are discussed in the text to facilitate comparison with other studies.

The analysis showed a decrease in exosome concentration from 4.4 × 10^10^ ± 3.1 × 10^10^ exosomes per mL in the control group to 3.7 × 10^10^ ± 3.6 × 10^10^ and 3.1 × 10^10^ ± 3.4 × 10^10^ in the PD and MSA groups, respectively (Supplementary Fig. 6a, online resource). Although these differences were statistically insignificant, because the same trend was found in the validation cohort, the exosome concentration was included in the final multivariate statistical model used to separate the groups (see below). The exosome concentrations did not correlate with disease duration in either group. Measurement of serum α-syn showed insignificant differences among the groups (Supplementary Fig. 6b, online resource).

**Fig. 1:**
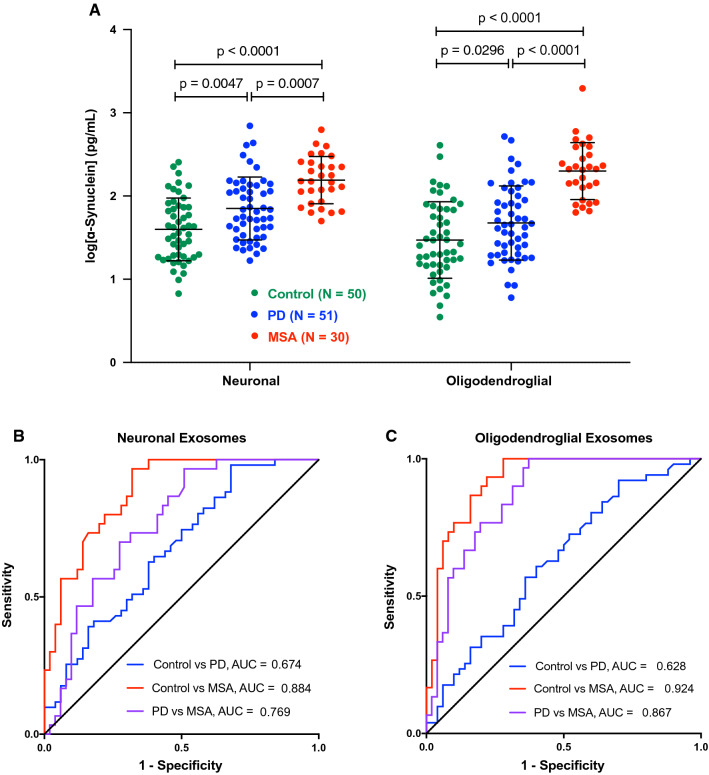
α-Syn concentration in putative neuronal and oligodendroglial exosomes differs significantly among the groups in the discovery cohort. **a** α-Syn concentrations were measured using ECLIA, log-transformed, and analyzed by a two-way ANOVA with post hoc Tukey test. The data are presented as mean ± SD. **b** ROC analyses of α-syn concentration in putative neuronal exosomes. **c** ROC analyses of α-syn concentration in putative oligodendroglial exosomes

In both the putative neuronal and oligodendroglial exosomes, α-syn increased in the order control < PD < MSA (Fig. 1a). The α-syn concentrations in the PD group (putative neuronal 107 ± 124 pg/mL, putative oligodendroglial 81 ± 104 pg/mL) were significantly higher than in the control (putative neuronal 58 ± 55 pg/mL, putative oligodendroglial 53 ± 73 pg/mL) and significantly lower than in the MSA group (putative neuronal 191 ± 131 pg/mL, putative oligodendroglial 286 ± 348 pg/mL). Interestingly, these results contradicted the observations of Jiang et al. [32] and Yu et al. [70] who reported lower α-syn concentrations in putative CNS-originating exosomes from patients with MSA compared to those with PD.

To evaluate the degree of overlap among the groups, we used ROC analyses. The separation between the control and PD groups was low in both the putative neuronal (AUC = 0.674, Fig. 1b) and putative oligodendroglial (AUC = 0.628, Fig. 1c) exosomes, in agreement with results reported previously by Shi et al. (AUC = 0.654 in putative neuronal exosomes from 215 control and 267 PD plasma samples) [61]. In contrast, high separation was found between the control and MSA groups, particularly in the putative oligodendroglial exosomes (AUC = 0.924, Fig. 1c). α-Syn concentration in putative oligodendroglial exosomes also provided better separation between the PD and MSA groups (AUC = 0.867, Fig. 1c) than in putative neuronal exosomes (AUC = 0.769, Fig. 1b).

 > 90% of the samples in the control and PD groups were from the UCLA PEG study in this cohort, precluding meaningful testing of a source effect. In the MSA group, 21 samples were from UCLA and 9 from NYU. Comparing the putative neuronal and oligodendroglial α-syn concentrations in the samples from these two sources we found in both cases that the concentrations in the NYU samples were significantly higher (Supplementary Table 1, online resource).

### The oligodendroglial: neuronal exosomal α-syn ratio improves the separation of PD from MSA

**Fig. 2:**
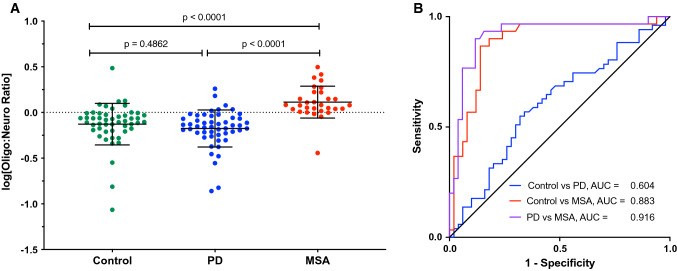
The ratio between α-syn concentrations in putative oligodendroglial and neuronal exosomes improves the separation between PD and MSA. **a** The ratio between the α-syn concentration in putative oligodendroglial and neuronal exosomes was calculated for each sample and log-transformed. The data are presented as mean ± SD. The dashed line indicates the cutoff at 0 (log1). *P* values were calculated using a one-way ANOVA with post hoc Tukey test. **b** ROC analysis of the oligodendroglial:neuronal exosome α-syn ratio

We asked next whether the known preference for deposition of α-syn in neurons in PD versus oligodendrocytes in MSA could further help distinguish between these groups, even though the measurement in our assay was of total, rather than aggregated α-syn. In agreement with the pattern of pathological α-syn deposition in the brain, in the PD group, the average α-syn concentration in the putative neuronal exosomes was higher than in the putative oligodendroglial exosomes, whereas the opposite was true in the MSA group (Fig. 1a). Therefore, we calculated the ratio between the α-syn concentration in the putative oligodendroglial and putative neuronal exosomes (oligo:neuro ratio) for each sample (Fig. 2a). In most cases, the ratio was as expected, < 1 for PD and > 1 for MSA, yielding AUC = 0.916 (Fig. 2b), corresponding to 90.0% sensitivity and 88.2% specificity. Importantly, unlike the total α-syn concentrations that differed significantly between the UCLA and NYU MSA samples, the difference between the average ratio values was insignificant (Supplementary Table 1, online resource), suggesting that the ratio may serve to remove variations among collection sites.

### Validation cohort

The samples used in our discovery cohort were collected from patients diagnosed clinically and not validated pathologically. To our knowledge, serum or plasma samples collected from living patients whose brains were analyzed pathologically after death are not available in sufficient numbers in any current biobank, especially considering that the samples cannot be > 5 years in storage. Therefore, we attempted to use samples collected post-mortem with a short post-mortem interval (PMI) from patients whose diagnosis was validated pathologically. Unfortunately, we found that in such samples, erythrocyte α-syn contaminated the signal and did not allow meaningful analysis of α-syn in putative CNS-originating exosomes (Supplementary Results, Supplementary Table 2, and Supplementary figs. 7, 8, online resources).

In the absence of pathologically validated samples, we obtained next a new set of samples to assemble a validation cohort. Most of these samples were obtained again from the UCLA PEG study (control and PD), the UCLA Clinical Neurogenomics Research Center (MSA), and the NYU Dysautonomia Center (PD and MSA). An additional major source of samples for this cohort was a biobank at Columbia University (control and MSA). Comparison of the two cohorts showed that the groups did not differ significantly in their composition in terms of sex, ethnicity, and age (Supplementary Table 3, online resource). A few samples from the UCLA Easton Center were included originally but later eliminated, because their storage time was > 5 years. Because this cohort contained both serum and plasma samples, an adjustment of the raw data was necessary to allow analysis of these samples together (Supplementary Results and Supplementary Fig. 9, online resources).

Similar to the discovery cohort, before immunoprecipitation, there was a trend toward reduced exosome concentration in the serum/plasma samples in the order: control > PD > MSA (Supplementary Fig. 10a, online resource). Interestingly, unlike the discovery cohort, α-syn concentration in the serum/plasma was substantially higher in the MSA group (486 ± 479 pg/mL) than in the control (187 ± 241 pg/mL) or PD (208 ± 183 pg/mL) groups (Supplementary Fig. 10b, online resource). As the majority of the MSA samples in this group were from Columbia University, we asked if differences among the sample sources might have accounted for the higher serum/plasma α-syn levels in this cohort. Comparison among the sources showed that despite the larger number of samples from Columbia University, the variability in these samples was lower than in the samples from UCLA or NYU, yet the differences among the groups were statistically insignificant (Supplementary Fig. 10c, online resource). The increased α-syn concentration in the MSA group could be partially attributed to the differences between the serum and adjusted plasma concentrations in these samples (Supplementary Fig. 9c, online resource). In addition, there was a larger fraction of MSA-P in the validation cohort (33.3%, including 4 samples with a mixed MSA-P/MSA-C diagnosis) compared to the discovery cohort (13.3%). On average, samples from patients diagnosed with MSA-C had lower serum/plasma α-syn concentrations (353 ± 251 pg/mL) than samples from patients with MSA-P or mixed diagnosis (547 ± 669,  *p*= 0.062, Student’s *t* test). Thus, the larger fraction of the latter in the validation cohort possibly also contributed to the increased concentration of serum/plasma α-syn in this cohort’s MSA group.

**Fig. 3:**
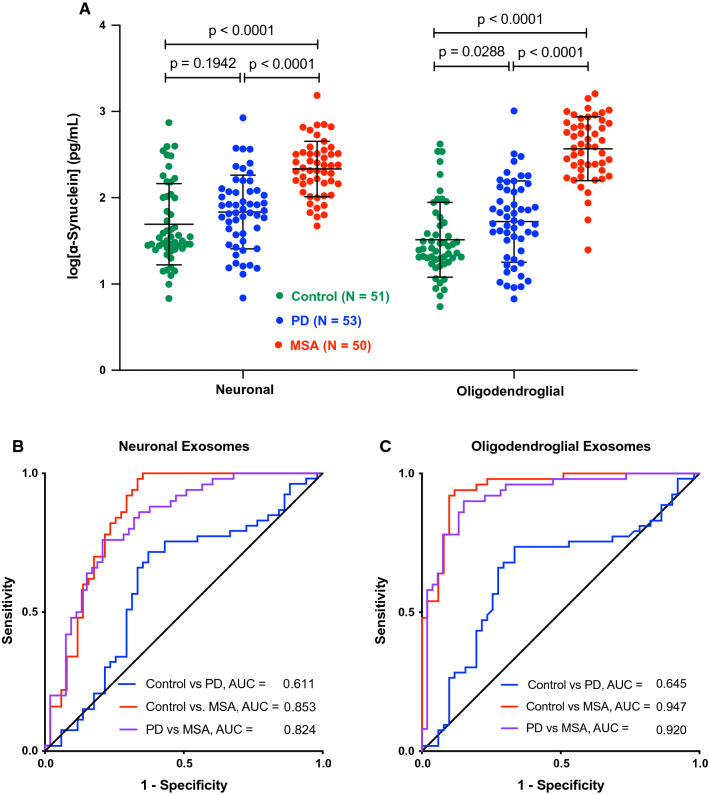
α-Syn concentration in putative neuronal and oligodendroglial exosomes differs among the groups in the validation cohort. **a** α-Syn concentrations were measured using ECLIA, log-transformed, and analyzed by a two-way ANOVA with post hoc Tukey test. The data are presented as mean ± SD. **b** ROC analyses of α-syn concentration in putative neuronal exosomes. **c** ROC analyses of α-syn concentration in putative oligodendroglial exosomes

Similar to the discovery cohort, the α-syn concentrations increased in the order: control < PD < MSA in both the putative neuronal and oligodendroglial exosomes, though the difference between the control and PD groups was statistically significant only for the latter (Fig. 3a). Higher concentrations of α-syn were observed in the immunoprecipitated exosomes from the MSA group in this cohort, possibly for the same reasons discussed above for serum/plasma α-syn. The average α-syn concentration in the PD group (putative neuronal 110 ± 136 pg/mL, putative oligodendroglial 96 ± 148 pg/mL) was closer to the control group (putative neuronal 96 ± 110 pg/mL, putative oligodendroglial 60 ± 91 pg/mL) and substantially lower than the MSA group (putative neuronal 284 ± 251 pg/mL, putative oligodendroglial 497 ± 360 pg/mL). Accordingly, the separation between the control and PD groups was moderate in both the putative neuronal (AUC = 0.611, Fig. 3b) and putative oligodendroglial (AUC = 0.645, Fig. 3c) exosomes, whereas the separation between the control and MSA groups, particularly in the putative oligodendroglial exosomes was high (AUC = 0.947, Fig. 3c). α-Syn concentration in putative oligodendroglial exosomes provided better separation between the PD and MSA groups (AUC = 0.920, Fig. 3c) than in putative neuronal exosomes (AUC = 0.824, Fig. 3b), mirroring the discovery cohort.

**Fig. 4:**
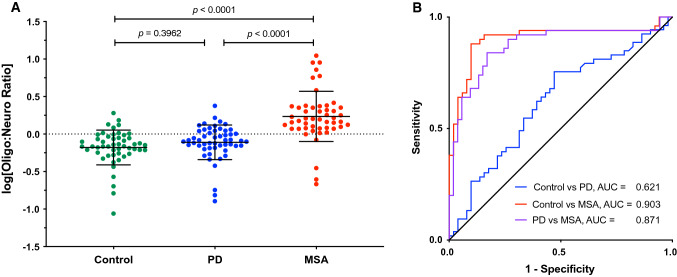
Validation of the separation between PD and MSA by the ratio between α-syn concentrations in putative oligodendroglial and neuronal exosomes. **a** The ratio between the α-syn concentration in putative oligodendroglial and neuronal exosomes was calculated for each sample and log-transformed. The data are presented as mean ± SD. The dashed line indicates the cutoff at 0 (log1). *P* values were calculated using a one-way ANOVA with post hoc Tukey test. **b** ROC analysis of the oligodendroglial:neuronal exosome α-syn ratio

Analysis of the oligo:neuro ratio (Fig. 4a) showed that the number of PD samples for which the ratio was < 1 was lower in this cohort (69.8%) than in the discovery cohort (88.2%), whereas the fraction of MSA samples for which the ratio was > 1 was similar in the validation cohort (90.0%) to the discovery cohort (86.7%). Accordingly, the separation between the PD and MSA groups was somewhat lower in the validation cohort, AUC = 0.871.

### Multivariable models for separation between PD and MSA

To further explore the degree to which the measured biomarkers could help improve distinguishing between PD and MSA, we tested several statistical models, including: (1) a multinomial logistic model with LASSO variable selection [16, 64]; (2) a linear discriminant model; (3) a classification tree model; and (4) a K-nearest-neighbor model. In each case, the model was trained on the discovery cohort and was challenged with selecting among the putative neuronal exosomal α-syn, putative oligodendroglial exosomal α-syn, oligo:neuro ratio, and serum/plasma exosome concentration of the combination providing the best separation among the groups in a ROC analysis. Because the first three parameters are interdependent, the models were expected to select two out of the three and fulfilled this expectation in all cases. The formula created by the model then was applied to the validation cohort to test to what extent the discrimination power could be reproduced.

All the models, except the K-nearest-neighbor model, which yielded poor accuracy (data not shown), provided similar results (Supplementary Table 4, online resource), yet the multinomial logistic model yielded the highest discrimination power. The model selected the putative neuronal exosomal α-syn, oligo:neuro ratio, and serum/plasma exosome concentration to create the discriminative formula, which in the discovery cohort separated the control and PD groups with AUC = 0.762, control and MSA with AUC = 0.961, and PD and MSA groups with AUC = 0.928. When the formula created by the model was applied to the validation cohort, it separated the control and PD groups with AUC = 0.610, control and MSA with AUC = 0.962, and PD and MSA groups with AUC = 0.902, corresponding to 89.8% sensitivity and 86.0% specificity.

A limitation of our study’s design was that it did not emphasize inclusion of early stage patients, in which the potential for misdiagnosis is highest. Most patients with PD in our study were 5–8 years from diagnosis, whereas the disease duration in the MSA group was mostly 3–5 years (Supplementary Fig. 11a, online resource). Examination of the biomarkers reported here in patients with early stage disease will be pursued in the future. Nonetheless, to test whether the main biomarker, the oligo:neuro ratio, can be detected in early stage disease or only develops at later stages, we tested whether this biomarker correlated with disease duration. Spearman analysis showed that the oligo:neuro ratio did not correlate with disease duration (Supplementary Fig. 11b–e, online resource), suggesting that it could be a useful biomarker already at the time of diagnosis.

Another potential limitation of the study is that the reference used for biomarker accuracy is the clinical diagnosis, which as discussed in the Introduction, is prone to error. Though validation of the biomarker’s accuracy through a neuropathological examination was not possible in most cases, toward the end of the study, we went back and checked whether any clinical diagnosis was validated after patients passed away or changed by the diagnosing clinicians. The data are presented in Supplementary Table 5 (online resource).

In the PD groups (both cohorts combined), one diagnosis was changed from PD to MSA-P, which was predicted correctly by the model. Two patients’ diagnosis was changed from PD to Parkinson’s disease dementia (PDD), of which one was predicted by the model to be MSA. No diagnosis was pathologically validated in this group. In the MSA group, the model predicted correctly the diagnosis of 11 out of 13 patients whose diagnosis with MSA, or in two cases MSA mixed with AD, was confirmed pathologically. In two cases, both of which were diagnosed clinically with probable MSA-C and later validated pathologically as MSA, the model predicted incorrectly a diagnosis of PD. These numbers are in agreement with the degree of sensitivity and specificity of the model described above.

In contrast to the diagnostic power of the biomarkers we measured, we did not find cross-sectional correlation with disease severity for any of the biomarkers, including measurements of motor deterioration (UPDRS-III, UMSARS, H&Y) or cognitive function (MMSE).

## Discussion

A diagnostic test for synucleinopathies is an urgent unmet medical need both for currently available treatments and for stratifying patients in clinical trials of new therapies. A blood-based test offers several advantages over current methodologies. It can be performed in typical clinics, does not require the use of radionuclides in the brain or a lumbar puncture, and is cost-effective. Analysis of biomarkers in CNS-originating exosomes allows comparison among exosomes from different cell types, offering an additional advantage compared to CSF analysis. Thus, for distinguishing between PD and MSA, we found that the ratio between α-syn concentration levels in putative oligodendroglial and neuronal exosomes was particularly useful, as most patients with PD were found to have higher α-syn levels in their putative neuronal exosomes, whereas those with MSA had higher levels in putative oligodendroglial exosomes, providing AUC = 0.871–0.916 in ROC analyses (Figs. 2, 4). In addition, the α-syn concentration in putative oligodendroglial exosomes itself was useful for separating the MSA from both the control and PD groups, in agreement with the lack of expression of α-syn in normal mature oligodendrocytes. The putative oligodendroglial exosomal α-syn concentration was significantly higher in MSA than in PD and separated between the PD and MSA groups with AUC = 0.867–0.920 (Figs. 1, 3). This result was in agreement with analyses of total brain α-syn, which showed that the concentrations, particularly in membrane-associated and insoluble extracts, are higher in MSA than in PD brains [58, 65].

Our study used methodologies first introduced by Shi et al. in PD research [61] and Goetzl and co-workers in the AD field [17], who used L1CAM to immunoprecipitate putative neuronal exosomes from serum or plasma. The high level of separation we achieved between the PD and MSA groups was facilitated by adding to this methodology immunoprecipitation of putative oligodendroglial exosomes using the specific oligodendrocyte marker MOG. We chose MOG, because it is a membrane-bound protein [7] and because specific commercial antibodies are available against its extracellular domain, which we predicted might be exposed on the surface of exosomes. Recently, Yu et al. have analyzed α-syn concentrations in putative oligodendroglial exosomes immunoprecipitated from plasma of patients with PD or MSA using a different oligodendrocyte marker, CNPase, and reported lower α-syn concentrations in the MSA groups compared to PD [70], in contrast to our findings (Figs. 1, 3). Although there are several methodological differences between the study by Yu et al. and our report, the most likely explanation for the apparent discrepancy in the results is the difference in the markers used for immunoprecipitating the exosomes. Although both MOG and CNPase are membrane-bound proteins expressed specifically by oligodendrocytes, CNPase is present on the cytosolic side of non-compact myelin [14, 66] and the intermembrane space of mitochondria [37], which may limit its presentation on the surface of exosomes. An alternative explanation is differences in the antibodies used for α-syn quantitation in the different studies. MSD does not share the identity of the capture and detection antibodies in the ECLIA kit we used, precluding making a side-by-side comparison.

Several other recent studies have reported biomarkers that could help distinguish between PD and MSA. Hansson et al. measured neurofilament light chain (NfL) in blood and CSF samples from patients with PD and other parkinsonian disorders, including PSP, corticobasal degeneration (CBD), and MSA [28] obtained from three different cohorts. As has been reported now in multiple studies [22, 43], a strong correlation was found between blood and CSF levels of NfL. NfL concentrations were elevated in PSP, CBD, and MSA compared to PD and healthy controls, allowing separation of the PD and MSA groups with AUC = 0.81–0.91 in the different cohorts [28]. PSP, CBD, and MSA samples had comparable levels of NfL. As PSP and CBD are tauopathies, α-syn levels in neuronal and oligodendroglial exosomes in these diseases are expected to be substantially lower than in MSA. Therefore, combining blood NfL with CNS-exosomal α-syn could allow separating MSA not only from PD but also from PSP and CBD.

More recently, Jiang et al. have reported that α-syn in putative neuronal exosomes isolated from the serum of patients with PD and several atypical parkinsonian syndromes was a useful diagnostic biomarker [32]. Although similar to most studies in this field, L1CAM was used for immunoprecipitation of the exosomes, they used a distinct kind of polymeric support from the one used by most groups, in an attempt to decrease non-specific binding of the exosomes to the beads themselves. They reported that α-syn concentrations in the putative neuronal exosomes from PD samples were higher than in MSA samples, in contrast to our findings. Although their study also included three cohorts, MSA samples were available in only one and their number was limited to 14 [32]. As with the study by Yu et al., the most likely explanation for the difference between their results and ours is the use of different reagents for immunoprecipitation. An important discovery in the study by Jiang et al. was that in addition to α-syn, clusterin concentrations in the putative neuronal exosomes also differed substantially among the disease groups. The combination of α-syn and clusterin allowed separating the small MSA group from the counterpart PD group with AUC = 0.94. This important discovery suggests that clusterin should be included in future studies aimed at validating these initial results.

An important recent study by Shahnawaz et al. found that PD and MSA could be separated with an overall sensitivity of 95.4% by applying protein misfolding cyclic amplification (PMCA) to CSF samples from 94 patients with PD and 75 patients with MSA (clinical diagnosis in all cases) [59]. The PMCA technique allows measurement of fibrillar α-syn with high sensitivity by signal amplification. Moreover, this and other studies (e.g., by Prusiner et al. [54]) have demonstrated that α-syn fibrils in PD and MSA form distinct conformational strains. Although our ECLIA measurements were of total α-syn, we cannot rule out that some of the differences we observed might have stemmed from different antibody reactivity toward α-syn strains in the PD and MSA samples. Future studies will tell if PMCA or similar techniques can be applied to α-syn in CNS-originating exosomes, which would alleviate the need for a lumbar puncture and offer the advantage of analyzing fibrillar α-syn in exosomes from different cell types, as was done here.

Though > 50 studies have demonstrated the utility of analyzing putative CNS-originating exosomes as a source of biomarkers to date, the ability to use anti-L1CAM antibodies for enriching putative neuronal exosomes by immunoprecipitation recently has come under scrutiny due to the existence of multiple forms of the protein, both soluble and membrane-bound, and because L1CAM is expressed also outside the brain in non-neuronal cells [47]. It is widely acknowledged that immunoprecipitation using L1CAM is expected to enrich CNS-neuronal exosomes, rather than to yield a pure population. Our data demonstrate the existence of small amounts of L1CAM in SEC fractions containing exosomes by the same method used by Norman et al. [47] (Supplementary Fig. 3, online resource). We also did not find high non-specific binding of α-syn to beads conjugated to the anti-L1CAM antibody 5G3 as found by Norman et al. for antibody UJ127. Our data strongly suggest that L1CAM is present on the surface of neuronal exosomes and can be used to immunoprecipitate CNS-neuronal exosomes. Nonetheless, as L1CAM is also expressed by other tissues and low levels of MOG RNA also have been reported in non-CNS cells, a presence of α-syn in exosomes immunoprecipitated using these two markers but originating outside the CNS could contribute to the data we observed. Such contribution would be expected to be different for PD, where peripheral α-syn deposition in the enteric system and the skin has been reported, and MSA, in which α-syn accumulation is thought to be limited to the CNS.

To our knowledge, a limitation of all the studies published to date using the strategy of biomarker analysis in putative CNS-originating exosomes, including our study, is the lack of validation of the cellular origin of the exosomes. We made multiple attempts to analyze potential markers, other than those used for immunoprecipitation, to validate the neuronal or oligodendroglial origin of the exosomes in our study, yet due to the very limited amounts of immunoprecipitated exosomes, the results were inconsistent. Future studies, likely using highly sensitive techniques, such as ECLIA or single-molecule array (Simoa), will be needed to achieve reliable validation of exosome origin.

Because MSA is a rare disease, obtaining large numbers of biofluid samples is challenging. Collection of samples from several sources, particularly in the case of MSA, limited the availability of consistent clinical measures. For example, clinics specializing in movement disorders or autonomic failure used the more common UMSARS scale to measure disease progression, whereas the ataxia-focused clinics used the SARA scale, limiting the ability to compare among these datasets. For cognitive evaluation, some of the providing clinics used the MMSE test, whereas others used MoCA. Although we converted the MoCA score to MMSE [36], these differences might have compromised our ability to obtain meaningful correlation between the biomarkers and cognitive decline, though a more likely explanation for the lack of correlation we observed was the fact that most of the samples were from patients with little or no cognitive decline.

We combined samples from several sources to obtain two independent cohorts that would allow validating the predictions made using the discovery cohort in the independent validation cohort. A conscious decision we made was to use relatively lenient inclusion criteria in anticipation of high heterogeneity not only in the patient population, but also among clinics and biobanks providing the samples, a prediction that proved to be correct (Supplementary Table 1, online resource). With these considerations in mind, we conducted extensive quality control experiments to ensure that the included samples reflected *bona fide* α-syn in putative CNS exosomes and that technical parameters, such as storage time or number of freeze–thaw cycles would not affect the final measurement. Although the diagnosis was validated pathologically only in a small number of cases (Supplementary Table 5, online resource), our data demonstrate that even in a heterogeneous collection of samples, disease stages, and disease types (e.g., MSA-C vs MSA-P), the model based on the objective biomarkers we measured corresponded to the clinical diagnosis of approximately nine out of ten patients in the PD and MSA groups. The diagnostic power was even higher for separating patients with MSA from healthy controls, whereas the separation of patients with PD from healthy individuals was moderate, as reported previously [61]. In the future, adding biomarkers, such as tau [60], pS129-α-syn [19, 35, 40, 69], and clusterin, and/or combining these biomarkers with measurements of oligomeric/aggregated α-syn, as was demonstrated by Shahnawaz et al. [59], holds promise for improving the diagnostic accuracy.

In conclusion, we tested and validated a blood-based diagnostic biomarker in two independent cohorts, which separates two related synucleinopathies, PD, and MSA, with high sensitivity and specificity. The biomarker is based on measurement of α-syn concentrations in putative neuronal and oligodendroglial exosomes isolated from patients’ serum or plasma. Additional validation in larger cohorts, and eventually in pathologically confirmed samples when those become available, may facilitate the use of this biomarker, potentially in combination with recently discovered ones, such as clusterin and fibrillar α-syn, for routine clinical diagnosis of these diseases.
